# Construction of a novel *Pichia pastoris* strain for production of xanthophylls

**DOI:** 10.1186/2191-0855-2-24

**Published:** 2012-04-25

**Authors:** José Miguel Araya-Garay, José M Ageitos, Juan A Vallejo, Patricia Veiga-Crespo, Angeles Sánchez-Pérez, Tomás G Villa

**Affiliations:** 1 Department of Microbiology, University of Santiago de Compostela, Santiago de Compostela, Spain; 2 School of Biotechnology, University of Santiago de Compostela, Santiago de Compostela, Spain; 3 Discipline of Physiology and Bosch Institute, University of Sydney, Sydney, NSW, 2006, Australia

**Keywords:** *Pichia pastoris*, Carotenoids, β-carotene, Astaxanthin

## Abstract

In this study, we used the yeast carotenogenic producer *Pichia pastoris* Pp-EBIL strain, which has been metabolically engineered, by heterologously expressing β-carotene-pathway enzymes to produce β-carotene, as a vessel for recombinant astaxanthin expression. For this purpose, we designed new *P. pastoris* recombinant-strains harboring astaxanthin-encoding genes from carotenogenic microorganism, and thus capable of producing xanthophyllic compounds. We designed and constructed a plasmid (pGAPZA-WZ) containing both the β-carotene ketolase (*crtW*) and β-carotene hydroxylase (*crtZ*) genes from *Agrobacterium aurantiacum*, under the control of the *GAP* promoter and containing an *AOX-1* terminator. The plasmid was then integrated into the *P. pastoris* Pp-EBIL strain genomic DNA, producing clone Pp-EBILWZ. The recombinant *P. pastoris* (Pp-EBILWZ) cells exhibited a strong reddish carotenoid coloration and were confirmed, by HPLC, to produce not only the previous described carotenoids lycopene and β-carotene, but also *de novo* synthesized astaxanthin.

## Introduction

Carotenoids are natural lipid-soluble pigments produced primarily by bacteria, algae and plants. These pigments are in part responsible for the wide variety of colors seen in nature. In some organisms, carotenoids such as β-carotene are modified with oxygen-containing functional groups, thus creating xanthophylls such as astaxanthin.

Astaxanthin is an abundant carotenoid found in marine animals, including salmonids and crustaceans (Miki et al. [1982]; Wade et al. [2005]) and is a commonly encountered keto-carotenoid in certain algae, many invertebrates and fish. The use of astaxanthin as colorant in aquaculture, especially as feed supplement in farmed trout, salmon and prawns, is necessary to obtain the red–pink coloration present in their wild counterparts, since neither fish nor prawns are capable of *de novo* carotenoid synthesis. Incorporation of astaxanthin into the fish and prawn feed not only increases their nutritional value, but also considerably enhances their appeal to customers and hence their commercial value.

Astaxanthin has attracted commercial interest not only in its role as a pigment, but also as a potent antioxidant capable of delaying aging and the onset of degenerative diseases in animals (Hix et al. [2004]; Kurihara et al. [2002]; Neuman et al. [2000]). Furthermore, epidemiological and experimental studies have suggested that astaxanthin also possesses anticarcinogenic and antitumor activities (Neuman et al. [2000]; Bertram & Vine [2005]; Kozuki et al. [2000]), hence astaxanthin’s relevance is progressively increasing in the pharmaceutical and cosmetic industries.

**Figure 1:**
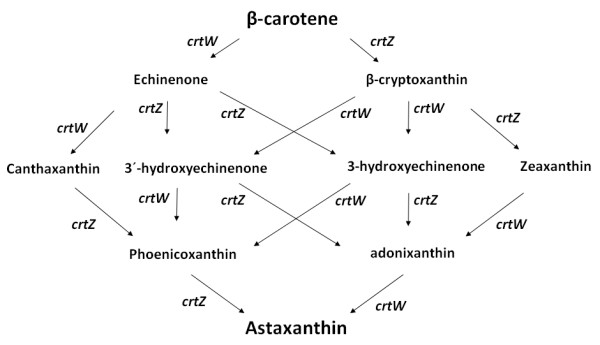
** Schematic diagram of astaxanthin biosynthetic pathways and possible intermediates in*Agrobacterium. aurantiacum*, modified from Misawa et al.**[1995.]

The cluster genes responsible for the synthesis of xanthophylls have been isolated from the marine bacterium *A. aurantiacum*. Analysis of its nucleotide sequence revealed five open reading frames, designated as genes *crtWcrtZcrtYcrtI*, and *crtB*, respectively (Misawa et al. [1995]) and functionally analyzed in *E*. *coli* (Misawa et al. [1995]). β-carotene ketolase (*crtW* genes) converts β-carotene to canthaxanthin, with echinenone as an intermediary step; whereas β-carotene hydroxylase (*crtZ* genes) mediates the conversion of β-carotene to zeaxanthin, via β-cryptoxanthin. As seen in Figure 1, the *crtW* and *crtZ* gene products, in combination, catalyze all the necessary steps for the conversion of β-carotene into astaxanthin (Figure 1). 

A variety of carotenoid biosynthesis genes that produce astaxanthin have been isolated from various sources, including the yeast *Xanthophyllomyces dendrorhous* (Johnson et al. [1980]), the green alga *Haematococcus pluvialis* (Bubrick [1991]), the gram-positive bacterium *Brevibacterium linens* (Krubasik & Sandmann [2000]), and the marine bacterium *Paracoccus haeundaensis* (Lee et al. [2004]), and the function of their gene products has been determined (Kurihara et al. [2002]; Krubasik & Sandmann [2000]; Armstrong et al. [1989]; Harker & Hirschberg [1998]; Harker & Hirschberg [1997]; Misawa et al. [1990]; Verdoes et al. [1999]).

Recombinant carotenoid biosynthesis was successful, by introduction and modification of heterologous carotenogenic genes, in originally non-carotenogenic yeasts, such as *Saccharomyces cerevisiae* (Lange & Steinbüchel [2011]; Ukibe et al. [2009]; Verwaal et al. [2007]; Yamamo et al. [1994]), both *S*. *cerevisiae* and *Candida utilis* (Misawa & Shimada [1998]), *C. utilis* (Miura et al. [1998]; Misawa & Shimada [1998]), *P. pastoris* (Araya-Garay et al. [2012]; Bhataya et al. [2009]), and the filamentous fungus *Mucor circinelloides* (Papp et al. [2006]).

In the present work, we successfully modified the carotenoid production of *P. pastoris* Pp-EBIL strain by incorporating in its genome the *crtW* and *crtZ* genes from the marine bacterium *A. aurantiacum*. This resulted in a recombinant *P. pastoris* which synthesized astaxanthin as well as pathway intermediates such as lycopene, β-carotene and canthaxanthin*.*

## Materials and methods

### Strains, plasmid and culture conditions

Plasmid pGAPZαA was purchased from Invitrogen Corporation (Carlsbad, CA, USA), whereas the β-carotene producer Pp-EBIL strain of *P. pastoris* was constructed as previously described (Araya-Garay et al. [2012]).

*P. pastoris* cells were grown in YPD medium supplemented with Zeocin (100 μg/mL; Invitrogen) and incubated at 30°C, in a rotary shaker at 200 rpm for 72 h. *Escherichia coli* TOP10 cells were grown in low salt LB medium at 37°C for 12 h, and clones containing plasmid pGAPZαA were selected by their Zeocin (25 μg/mL) resistance. pGAPZαA* (a mutant pGAPZαA missing an *Avr*II site) was generated by site-directed mutagenesis (Araya-Garay et al. [2012]). Genes *crtW* and *crtZ* were amplified from the plasmid pAK96K (Misawa et al. [1995]), which harbors both the *A*. *aurantiacum crtW* (β-carotene ketolase) and *crtZ* (β-carotene hydroxylase) genes, and was shown to mediate the conversion of β-carotene into astaxanthin in recombinant *E. coli* cells. This plasmid was a gift from Prof. Misawa (Research Institute for Bioresources and Biotechnology, Ishikawa Prefectural University, Japan). Amplification of the above mentioned genes was carried out using 5′ primers that contained a restriction *Sfu*I site, followed by an optimized Kozak consensus sequence (ATGG), as well as a start codon, and a 3′ primer containing an *EcoR*I restriction site (Table 1). All DNA ligations were carried out with T4 DNA ligase (New England BioLabs, Beverly, MA, USA), as recommended by the manufacturer. After DNA ligation, the plasmids were transformed into chemically-competent *E. coli Top* 10 “One shot” (Invitrogen), and grown on low salt Luria–Bertani media (0.5% Yeast extract, 1% Tryptone, 0.5% NaCl) plates containing 25 μg/mL Zeocin. The plates were then incubated overnight at 37°C and recombinant colonies selected and grown overnight in low salt LB media containing 25 μg/mL Zeocin. 

**Table 1:** Oligonucleotide primers used in this study for either PCR-amplification or DNA sequencing

**Primer name**	**Primer sequence (5′-3′)**	**Application**
Aa-crtW Forward	5′ **AACTATTTCGAAACGATGG**CACATGCCCTGCC 3′	PCR
Aa-crtW Reverse	5′**GGAATTC**TCAGCGGTGCCCCC 3′	PCR
Aa-crtZ Forward	5′ **AACTATTTCGAAACGATGG**CAAATTTCCTGATCG 3′	PCR
Aa-crtZ Reverse	5′ **GGAATT**CTCACGTGCGCTCCTGC 3′	PCR
pGAP Forward 1	5′ GTCCCTATTTCAATCAATGAA 3′	Sequencing
pGAP Forward 2	5′ AGATCTTTTTTGTAGAAATGTC 3′	Sequencing
AOX-1 Reverse	5′ GCAAATGGCATTCTGACATCC 3′	Sequencing

The Graphical Codon Usage Analyser 2.0 (Fuhrmann et al. [2004]) was used for differential codon usage analysis.

### Construction of carotenoid expression vectors

**Figure 2:**
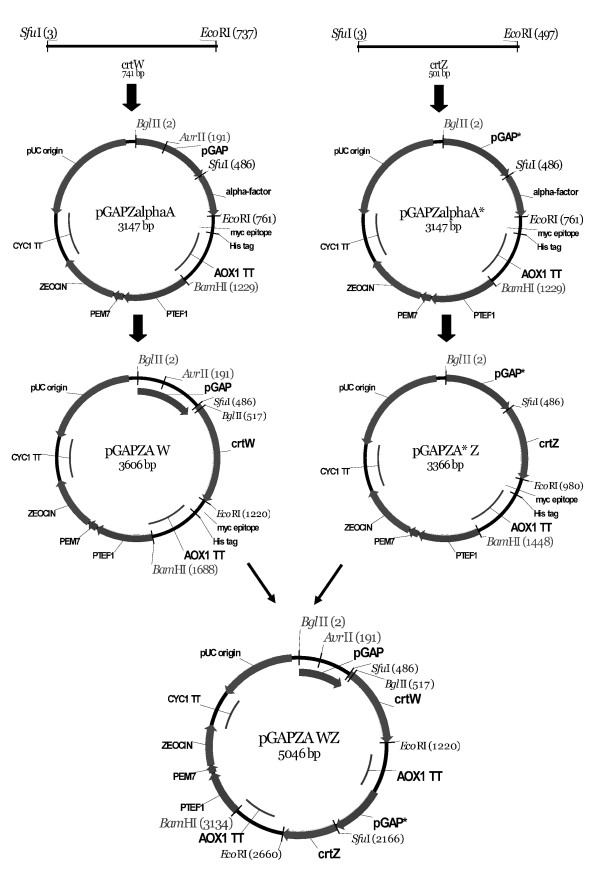
Construction of plasmids pGAPZA-W and pGAPZA*-Z, containing*ctrW*and*ctrZ*genes, respectively. The expression plasmid pGAPZA-WZ, coding for both *ctrW* and *ctrZ*, was used to transform *P. pastoris* Pp-EBIL cells. Plasmid pGAPZA* represents a plasmid pGAPZA in which the *Avr*II restriction site has been eliminated by mutation.

The DNA coding for *crtW* was inserted into the *Sfu*I and *EcoR*I restriction sites of plasmid pGAPZαA, and the same restriction sites were used for inserting *crtZ* DNA into pGAPZαA*. The resulting expression vectors were denominated pGAPZA-W and pGAPZA*-Z, respectively and both plasmids lacked the alpha factor (Figure 2). The *BamH*I–*Bgl*II DNA fragment from pGAPZA*-Z was subcloned into the *BamH*I site of plasmid pGAPZA-W to generate the pGAPZA-WZ expression vector (Figure 2). All plasmids constructed in this study were subjected to DNA sequencing before use and are shown in Table 2.

**Table 2:** Summary of the DNA plasmids used and/or constructed in this study

**Plasmid name**	**Description**	**Source or reference**
pGAPZαA	Integrative plasmid for *P.* pastoris (Zeo^R^)	(Invitrogen)
pGAPZαA*	pGAPZαA plasmid without *Avr*II site	(Araya-Garay et al. [2012])
pGAPZA-W	*crtW* gene cloned in pGAPZA without α factor	This study
pGAPZA*-Z	*crtZ* gene cloned in pGAPZA* without α factor	This study
pGAPZA-WZ	*crtW* and *crtZ* genes cloned in pGAPZA	This study

### Plasmid transformation

Plasmid pGAPZA-WZ was linearized with the restriction enzyme *Avr*II (New England BioLabs) and transformed into electrocompetent *P. pastoris* Pp-EBIL cells by electroporation, using a Bio-Rad Micropulser (Bio-Rad Laboratories, Inc Hercules, CA) as described previously (Araya-Garay et al. [2012]). Recombinant *P. pastoris* cells were then selected on YPDS (1% yeast extract, 2% peptone, 2% glucose, and 1 M sorbitol) plates, supplemented with Zeocin (200 μg/mL). The plates were incubated at 30°C until colonies were visible (48–72 h), transferred to room temperature, and incubated for a further 48–72 h. Gene integration into the *P. pastoris* genome was analyzed by PCR, using *P. pastoris* genomic DNA extracted with the Master Pure Yeast DNA Purification Kit (Epicentre Biotechnologies, Madison, WI, USA).

### Yeast culture and harvest

Red (Pp-EBILWZ) *P. pastoris* colonies, obtained in YPDS agar plates, were selected and grown, for 72 h at 30°C, with shaking at 200 rpm, in 100 to 500 mL of YPD (yeast extract 1%, peptone 2%, and glucose 2%) media containing 200 μg/mL Zeocin. The cell culture was then harvested, washed with distilled water, centrifuged and lyophilized for 48 h at 0.1 mbar in a Telstar Cryodos Lyophilizer.

### Carotenoid extraction

Prior to carotenoid extraction, 50 mg of lyophilized yeast cells were incubated in 3 mL of DMSO, pre-warmed at 55°C for 30 min, with strong shaking for 1 min, and then maintained for an extra 30 min without shaking (Dos Santos et al. [2011]). Residual cell-suspension, from each of the above treatments, was extracted with 10 mL of acetone and vortexed for 5 min at 4°C. Extracts were then combined with 5 mL of hexane and 1 mL of 0.1 M phosphate buffer, followed by vortexing for 30 s and centrifugation at 3000 g for 10 min. This extraction procedure was repeated until both the supernatant and residual cell pellet were colorless. The crude solvent extract thus obtained was then evaporated, under a stream of N_2_ flow, and kept at −80°C until high performance liquid chromatography (HPLC) analysis. All above treatments were carried out on ice and under dim light conditions, to prevent photo-degradation, isomerization and structural carotenoid changes.

### HPLC analysis of carotenoids

Carotenoid samples were prepared for HPLC by dissolving their cryo-preserved dry extracts in 2 mL of chlorophorm:metanol:acetone (3:2:1, v:v:v) and filtering them through polycarbonate 0.22 μm membranes. HPLC was carried out on a C_30_ carotenoid column (250 mm x 4,6 mm, 5 μm; YMC Europe), as previously described (Araya-Garay et al. [2012]). Carotenoids were identified by comparing their HPLC retention time and color with commercial standards. The β-carotene and astaxanthin standards were obtained from Sigma-Aldrich (Madrid, Spain). For each elution, a Maxplot chromatogram was obtained, displaying the carotenoid elution profile and its corresponding maximum absorbance wavelength. Qualitative analyses were carried out by comparing the carotenoid profiles obtained with the retention times for the β-carotene and astaxanthin standards.

## Results

### Construction of expression plasmids

The coding regions for genes *crtW* and *crtZ, from A*. *aurantiacum,* were PCR amplified from the plasmid pAK96K (Misawa et al. [1995]). The PCR products were then subcloned into the *Sfu*I and *EcoR*I sites of pGAPZαA, an expression vector containing a constitutive *GAP* promoter and an *AOX-1* terminator, generating plasmids pGAPZA-W and pGAPZA*-Z (without *Avr*II site) (Figure 2). The *BamH*I–*Bgl*II fragment from plasmid pGAPZA*-Z was then subcloned into the *BamH*I restriction site of plasmid pGAPZA-W and the resulting construct integrated into *P. pastoris* Pp-EBIL DNA genome by recombination events. Finally, plasmid pGAPZA-WZ (5046 bp) was designed, constructed and introduced into the yeast *P. pastoris* to produce astaxanthin (Figure 2).

### Characterization of the *Pichia pastoris* recombinant clones

**Figure 3:**
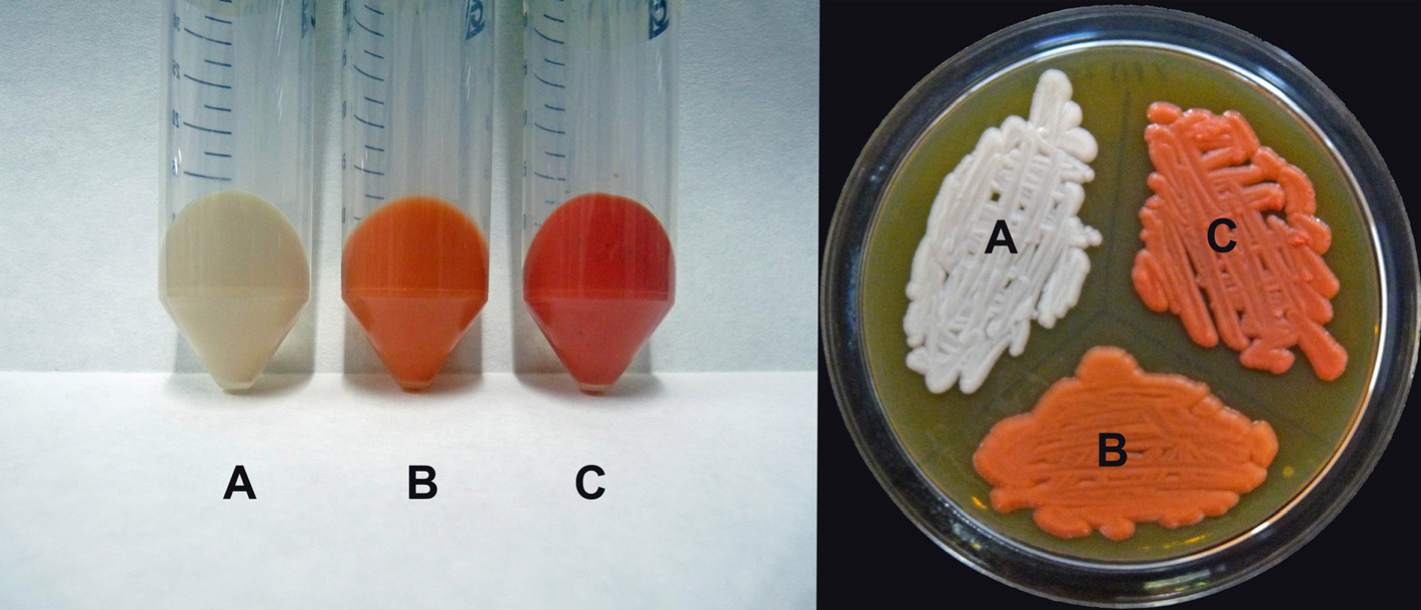
** Photographs of*P. pastoris*wet cell pellets (left) and agar plates (right). (A)** Non-transgenic culture; **(B)** Recombinant Pp-EBIL cells producing lycopene and β-carotene; **(C)** Recombinant Pp-EBIL strain harboring the plasmid pGAPZA-WZ and producing lycopene, β-carotene and astaxanthin. The reddish color corresponds to the carotenoids produced by the transgenic cultures.

The wild type *P. pastoris* X-33 yeast cells, shown in Figure 3A, display the typical white color characteristic of this strain. On the other hand, the Pp-EBIL recombinant strain, we used as the base for our transformation, shows an orange color (Figure 3B) typical of a strain producing lycopene and β-carotene (Araya-Garay et al. [2012]). Finally, integration of the plasmid pGAPZA-WZ into the Pp-EBIL genome resulted in yet another visible change in the color of the recombinant cells. The red cultures thus obtained (Figure 3C) are what will be expected from cells capable of *de novo* production of the carotenoid astaxanthin. The Pp-EBILWZ recombinant cells were confirmed, by PCR analyses, to contain the six recombinant genes we transformed integrated in their genomic DNA. 

### HPLC analyses of carotenoids

**Figure 4:**
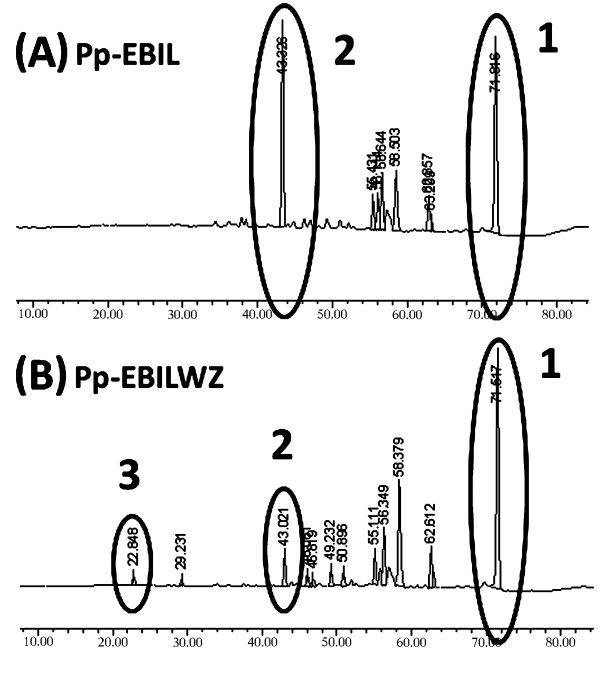
** MaxPlot chromatograms of cell extracts from*P. pastoris*: (A)** Pp-EBIL strain, producing lycopene (peak 1) and β-carotene (peak 2); **(B)** Pp-EBIL strain harboring plasmid pGAPZA-WZ and producing lycopene (peak 1), β-carotene (peak 2) and astaxanthin (peak 3).

To further confirm the nature and composition of the carotenoids produced by the red recombinant Pp-EBILWZ cultures, the photochromic compounds were extracted from the lyophilized cells and analyzed by high resolution liquid chromatography, coupled to a photodiode array detector (HPLC-PDA). These analyses revealed that the Pp-EBIL strain, carrying the plasmid pGAPZA-WZ, did indeed synthesize astaxanthin and this was accompanied by the accumulation of biosynthesis precursors, such as lycopene, β-carotene and a small amount of canthaxanthin, but no zeaxanthin was detected (Figure 4). The astaxanthin concentration produced by the cultures was 3.7 μg per g of cells (dry weight).

**Figure 5:**
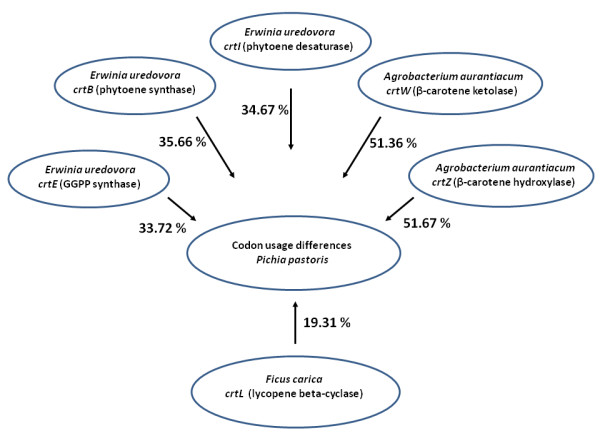
**Differences in synonymous codon usage in*Pichia pastoris*genome, as compared to*Erwinia uredovora ctrE*,*ctrB*and*ctrI*genes,*Agrobacterium aurantiacum ctrW*and*ctrZ*genes, and*Ficus carica crtL*gene.**

It has now been known for some time (Komar et al. [1999]) that synonymous codon substitutions may not always be silent, they can change protein structure and function and can be responsible for low expression of heterologous proteins (recently reviewed by (Angov [2011])). To investigate whether the low astaxanthin production by our recombinant Pp-EBIL strain could be attributed to differences in synonymous codon usage between expression and natural hosts, we used the Graphical Codon Usage Analyser 2.0 (Fuhrmann et al. [2004]) to compare codon usage by the expression host *(P. pastoris*)*,* the two natural hosts *(E. uredovora* and *A. aurantiacum*) and the fig tree (*F. carica*). As shown in Figure 5, the codon usage by *E. uredovora* is markedly different (differences ranging from 33.72 to 35.66%) from that of *P. pastoris.* The difference is even more marked (~51%) with *A. aurantiacum*, whereas the fig tree appears to be more closely related to our expression host (only 19.31% differences). 

From the Figure 5 results, it appears that the low astaxanthin production by our recombinant Pp-EBIL strain could indeed be due to differences in synonymous codon usage between *P. pastoris* and the recombinant genes natural hosts, and this is an area we are currently investigating.

## Discussion

Although *S. cerevisiae* and *P. pastoris* share considerable genetic similarity that has enabled expression of similar genes and compatibility between vectors, *P. pastoris* has a strong preference for respiratory metabolism. This means that the latter can grow at high cell densities without the accumulation of ethanol, an event that usually occurs in *S. cerevisiae* (Cereghino et al. [2002]) and hinders culture growth and hence protein production. Other advantages of using *P. pastoris* for heterologous protein expression reside on the simplicity of this system, the availability of strong promoters to drive gene expression, and the ability of this system to perform eukaryotic post-translational modifications at low cost (Cregg et al. [2002]; Lin Cereghino & Cregg [2000]).

On the other hand, yeasts have several cellular organelles which are physically separated from other cellular components by membrane structures (Karpichev & Small [2000]). The heterologously expressed six enzymes were designed to be randomly distributed in *P. pastoris,* and both cellular and cytoplasmic membranes can be putative locations for membrane-bound enzymes to settle in (Bhataya et al. [2009]). Therefore, since other yeasts such as *S. cerevisiae* and *X. dendrorhous* have similarity on the structural constrains of the cells and they have higher levels of astaxanthin production, we believe that the structural constrains of *P. pastoris* is it not a limit factor for astaxanthin production.

In the present work, we have succeeded in constructing genetically-stable astaxanthin-producing *P. pastoris* strains (Pp-EBILWZ). We achieved this by introducing the carotenogenic genes *crtW* (β-carotene ketolase) and *crtZ* (β-carotene hidroxylase) into a β-carotene-producing *P. pastoris* strain (Pp-EBIL) we previously engineered (Araya-Garay et al. [2012]) under the control of a *GAP* promoter.

DNA integration into a *GAP* locus requires linearization of the expression vectors with *Avr*II, and there is a recognition site for this restriction enzyme within the coding region of the *GAP* promoter. To avoid this complication, we removed, by site-directed mutagenesis, the *Avr*II restriction site within the pGAPZαA plasmid thus generating the silent-mutated plasmid pGAPZαA*. This plasmid was further modified by addition of the two *crt* genes required for the synthesis of astaxanthin from β-carotene (Figure 2), giving rise to the integrative plasmid we named pGAPZA-WZ. Recombinant plasmid pGAPZA-WZ was then integrated into Pp-EBIL genomic DNA, resulting in the production of yeast cells with a red coloration (Figure 3).

To determine the composition of the carotenoids produced by Pp-EBILWZ, this strain was grown for 3 days in liquid culture containing Zeocin (200 μg/mL), and the carotenoid content in the yeast cells analyzed by HPLC.As shown in Figure 4, our recombinant *P. pastoris* strain was indeed capable of synthesizing new xanthophylls, but its astaxanthin production level was below its β-carotene production. Additionally, the accumulation of astaxanthin metabolic intermediates indicates that the flux through the carotenogenic pathway was not fully efficient. The astaxanthin yield we obtained from our recombinant yeast is lower than those previously reported for heterologous astaxanthin production in *C. utilis* (Miura et al. [1998]) with the amounts of 400 μg per g of cells (dry weight) and *S. cerevisiae* (Ukibe et al. [2009]) with 29 μg per g of cells (dry weight); although it is very close to the yield obtained in *M. circinelloides* (Papp et al. [2006]) with 3 μg per g of cells (dry weight). Whereas in other microorganisms such as *X. dendrorhous* and *H. pluvialis* a significantly higher level of production are observed (120 μg and 114 μg per g of cells [dry weight], respectively). It should be noted that Pp-EBIL cells accumulated more β-carotene (339 μg per g [dry weight] of cells) than the total amounts of astaxanthin and β-carotene in the wild-type cells of *X. dendrorhous* (270 μg per g [dry weight] of cells). The Pp-EBILWZ had an additional drawback, as its growth was slower than that of the Pp-EBIL strain it originated from.

From the results shown in Figure 5, it appears that the low astaxanthin production by our recombinant Pp-EBILWZ strain could be due to differences in synonymous codon usage between *P. pastoris* and the recombinant genes natural hosts. This codon usage appears to be related to the intracellular availability of each tRNA, whose concentration is relatively proportional to the frequency of its complementary codon coding sequences population. This suggests that the speed of translation and, therefore, carotenoid protein production, may be limited and our recombinant strain cannot achieve high protein expression level for all of the six foreign genes the cells host. It must also be taken into account that the six recombinant genes are all members of the same pathway and are under the same *GAP* promoter. This could cause metabolic stress in the yeast cells, by limiting the availability of transcription factors required for proper expression of all the pathway proteins. Metabolic overload could be the cause of the slowing down of the cell growth observed in Pp-EBILWZ, as compared with the two strains (Pp-EBIL and *P. pastoris* X-33) it originates from.

However, based on the published strategies for improvements in the production of carotenoids described for other organisms, either by over-expression of genes, codon usage optimization or modification of gene members of the pathway, we believe that it is possible to increase our current astaxanthin production levels in *P. pastoris* to an industrially-relevant yield*.* One approach worth considering is that reported by Verwaal et al. (Verwaal et al. [2007]) and Yuan et al. (Yuan et al. [2006]), using mutated cultures and special fermentation conditions in large volumes. This strategy has worked well for *X. dendrorhous,* resulting in a marked increase in astaxanthin production (An et al. [1989]).

In conclusion, the results shown here indicate that it is indeed feasible to biosynthesize astaxanthin using the β-carotene-producing *P. pastoris* strain (Pp-EBIL) here described, although further investigation is required in order to improve the protein yield. This represents a further step in recombinant carotenoid production, and carotenoids, astaxanthin in particular, play an important role in the aquaculture industry and their addition into the fish and prawn feed not only increases their nutritional value, but also considerably enhances their appeal to customers and hence their commercial value. Additionally, there is increasing concern about food security, in particular fish and sea food, and aquaculture is progressively replacing shortages in fish catches, caused by overfishing, pollution, climate change and other insults to the marine habitats.

## Competing interests

The authors declare that they have no competing interests.
